# Validation of the Assessment of Rehabilitation Needs Checklist in a Swedish cancer population

**DOI:** 10.1186/s41687-024-00818-5

**Published:** 2024-12-05

**Authors:** Emma Ohlsson-Nevo, Maria Fogelkvist, Lars-Olov Lundqvist, Johan Ahlgren, Jan Karlsson

**Affiliations:** 1 Department of Surgery, Faculty of Medicine and Health, Örebro University, Örebro, Sweden; 2 University Health Care Research Center, Faculty of Medicine and Health, Örebro University, Örebro, Sweden; 3 Department of Oncology, Faculty of Medicine and Health, Örebro University, Örebro, Sweden; 4 Regional Oncological Centre Uppsala-Örebro, Uppsala, Sweden

**Keywords:** Checklist, Cancer rehabilitation, Validation, Hälsoskattning, Survey

## Abstract

**Background:**

Assessment of Rehabilitation Needs Checklist (ARNC), has been developed to assess rehabilitation need in cancer patients and is recommended by the Confederation of Regional Cancer Centres in Sweden, known as Hälsoskattningen. The aim of the study was to test the reliability and validity of the ARNC, mainly by comparing it with the Distress thermometer and EORTC QLQ-C30.

**Methodology:**

A sample of 993 persons identified in the Swedish cancer register. The study participants were diagnosed with cancer in 2021 in the Mid Sweden region. The psychometric methods tested reliability and validity including factor analysis.

**Results:**

The response rate was 38%. The test-retest analysis showed that ICC was 0.80 or higher for 12 of the ARNC items. A strong or modarete correlation between ARNC and the other instruments was found in all functional scales and for most items. CFA of the 13-item two-factor model showed a RMSEA value of 0.04, CFI and TLI values of 0.97 and 0.96, and a SRMR value of 0.05, indicating a satisfactory model fit.

**Conclusion:**

The evaluation of the ARNC suggests that it is an acceptable and reliable screening instrument for detecting symptoms and signs indicating a possible need of rehabilitation. The medium to strong correlations between ARNC items and the EORTC QLQ- C30 items and scales suggest that ARNC could be an alternative also for research purposes when a shorter and less comprehensive instrument is needed. The simple design could be an advantage as it lowers the burden on cancer patients.

**Supplementary Information:**

The online version contains supplementary material available at 10.1186/s41687-024-00818-5.

## Background

The number of cancer survivors who need to recover and return to life after cancer treatment is growing. Cancer and its treatments can have a negative impact on quality of life (QoL) [1–6], both during and after completion of treatment although cancer rehabilitation can reduce symptoms, enhance recovery and lead to better health and wellbeing [7]. Rehabilitation needs for an individual diagnosed with cancer includes both physical and psychological aspects of well-being. Since the diagnosis cancer can affect the patient’s wellbeing even before treatment have started, it is important that the patients’ needs should be assessed by health professionals throughout the cancer trajectory and rehabilitation should be tailored to the individual. It cannot be assumed that the patients themselves have knowledge of which interventions are adequate and available to reduce their signs and symptoms.

Screening instruments used in the clinical setting to facilitate the assessment of patients’ rehabilitation needs include questionnaires with one item for each health problem as well as questionnaires that use multiple items to obtain a scale score. Single-item questionnaires can provide a quick profile [8] of a patient’s health status, while multi-item scales are considered more stable, reliable, and precise, and suitable for research purposes [9]. Well-known instruments used in cancer care are the Distress Thermometer (DT) [10] and the European Organization for Research and Treatment of Cancer (EORTC) Quality of Life Questionnaire (QLQ-C30) [10]. These instruments’ main purpose is not assessing the need of rehabilitation but Health related Quality of Life and distress. A major disadvantage of the QLQ-C30 for screening purposes is that recalculation is needed to obtain scale scores which can be a challenge in clinical care and therefore a barrier of its use. The DT mainly measures distress on a scale of 0–10, while dichotomous response options (yes/no) are used for the instrument’s 35-item “problem list”.

Clinical oncology nurses in Sweden have requested a simple instrument to assess the rehabilitation needs of their patients as well as the possibility to assess progression over time. This request has led to the development of the Assessment of Rehabilitation Needs Checklist (ARNC), an instrument for Patient Reported Outcome Measures. The ARNC was developed through a literature review and interviews with clinicians and patients. Version 1 of the ARNC includes the recommended core set of symptoms to be measured in adult cancer care [11], namely fatigue, insomnia, pain, anorexia, dyspnea, cognitive problems, anxiety, nausea, depression, sensory neuropathy, constipation, and diarrhea. Additional items, included after discussion with oncology team members, are existential thoughts, appearance, physical activity, sexuality, family/relations, economy, and work/occupation. The ARNC has been validated locally with cognitive interviews and psychometric methods. It has been used in clinical settings in Sweden since 2016 and is recommended by the Confederation of Regional Cancer Centres in Sweden (Nationellt vårdprogram Cancerrehabilitering, cancercentrum.se). The current version, ARNC version 2, includes two additional items, *Balance and Addiction.* Addiction is added *since* smoking can reduce the efficiency of the cancer treatment [12] and alcohol can affect compliance with treatment. Problems with balance can lead to falls and fractures that need to be prevented. The final stage of development is to test the performance of the current version and compare it with other well-known instruments for assessing cancer rehabilitation needs. This study is part of a larger project with the purpose of following cancer patients’ rehabilitation needs during12 months.

The aim of this study was to test the reliability and validity of the ARNC, mainly by comparing it with the DT and QLQ-C30.

## Methods

### Study design

A postal survey was conducted in the health care region of Mid-Sweden.

### Power calculation

A sample of 200–400 participants is considered adequate for psychometric evaluation [13]. Based on previous surveys with similar sample of cancer patients, we expected a response rate of 50–60% [14, 15] and about 20% loss in follow-up, which means that a total sample of 1000 persons was needed.

### Setting

#### Study sample

A sample of 1000 persons diagnosed with the ten most common cancer diagnoses was identified in the Swedish Cancer Register. Inclusion criteria were patients who had been diagnosed with cancer in a hospital in the Mid-Sweden Health Care Region between March and June 2021 and presumably being affected by becoming a cancer patient and begin to have side effects from their treatments. An equal number of women and men were drawn from the register for each diagnosis except prostate and breast cancer where we included a larger number. Seven persons from the register were deceased and the final sample consisted of 993 persons (Table 1).

**Table 1:** Characteristics of the study sample

Total	*n* = 382
*Gender*, *n* (%)	
Men	209 (54.9)
Women	172 (45.1)
*Age*	
Mean (SD)	70.4 (9.5)
Range	22–97
*Age groups*, *n* (%)	
20–29	2 (0.5)
30–39	3 (0.8)
40–49	14 (3.7)
50–59	36 (9.5)
60–69	87 (22.8)
70–79	181 (47.5)
80+	58 (15.2)
*Civil status*, *n* (%)	
Married/cohabiting	259 (69.6)
Single	101 (27.2)
Other	12 (3.2)
*Education*, *n* (%)	
Mandatory	113 (30.0)
High school	95 (25.2)
University	138 (36.6)
Other	31 (8.2)
*Current occupation*, *n* (%)	
Working	63 (16.7)
Sick leave	15 (4.0)
Retirement pension	282 (74.8)
Other	17 (4.5)
*Cancer diagnosis*, *n* (%)	
Breast	89 (23.3)
Bladder	20 (5.0)
Prostate	89 (23.3)
Kidney	17 (4.4)
Pancreas	13 (3.4)
Lymph & Blood	39 (10.2)
Skin (malign melanoma)	30 (7.8)
Lung	32(8.4)
Rectum	16 (4.1)
Colon	36 (9.4)

### Data collection

The questionnaires, along with a form for informed consent and a prepaid return envelope, were distributed through the mail in March 2022. After the completed questionnaire was returned a retest questionnaire was posted to respondents. The retest was carried out online or on paper according to the respondent’s preferences.

The study was approved by the Regional Ethical Review Board in Lund, Sweden (Dnr 2021-05567-01).

### Questionnaires

The *Distress Thermometer* (DT), version 1, is a self-assessment instrument comprising a global distress rating and a “problem list” containing 35 single-item questions on cancer-related problems [10]. The global rating measures distress during the last week on a vertical numerical scale of 0 to 10, with a design resembling a thermometer [16]. The problem list is grouped into five categories concerning practical, physical, emotional, and family-related problems, and existential/religious concerns, with yes/no response alternatives [10]. The DT has been validated in several countries [17] including Sweden [18, 19] and is recommended by the Confederation of Regional Cancer Centres in Sweden (Nationellt vårdprogram Cancerrehabilitering, cancercentrum.se) as one of the instruments that can be used for assessing cancer rehabilitation needs.

The *European Organization for Research and Treatment of Cancer Quality of Life* Questionnaire *(EORTC QLQ-C30)* [20] is a widely used instrument for assessing general health-related QoL (HRQoL) in cancer patients, and is frequently used in cancer research [13]. The instrument comprises both single items and multi-item scales. It includes five functional scales, three symptom scales, and a global health status scale. All scales and single items are transformed to a 0–100 scale. Higher functional scores indicate better HRQoL, while higher symptom values indicate more problems.

The *Assessment of Rehabilitation Needs Checklist (ARNC)*, version 2, comprises 21 single-item questions about cancer-related symptoms and problems measured on a 4-point response scale where 1 = not a problem, 2 = a small problem, 3 = a troublesome problem, and 4 = a very troublesome problem. ARNC is a single item instrument with one item for each problem, without forming multi item scales or domains. The purpose of ARNC is to assess cancer rehabilitation needs and takes about 5 min to complete.

For this study the item responses were transformed to a 0–100 score to enable comparisons with the other instruments used in the study. Transformation is not necessary in regular clinical use but can be useful in research as many instrument scores are 0-100.

### Statistical analysis

Means and standard deviations (SDs) are presented for continuous variables, and relative frequencies for categorical variables. Two group comparisons were performed using the *t*-test for continuous data and Mann-Whitney test for ordinal data. Mean values are presented to allow comparison with other studies, although the data are ordinal level. The effect size (ES) was calculated as the mean difference divided by the pooled SD. Effect size criteria are: small 0.20–0.49, medium 0.50–0.79, and high ≥ 0.80 [13].

### Psychometric methods

#### Test–retest reliability

To test the reliability of the ARNC, the baseline measurement was compared with a retest after 2 weeks. The hypothesis was that the respondents’ responses would not change between these measurements. To calculate the consistency of measurements, the intraclass correlation coefficient (ICC) was calculated [21]. This ranges between 0 and 1 and a higher value indicates better reliability. A coefficient ≥ 0.7 is generally considered adequate [4].

#### Completeness of data

Completeness of data was evaluated by calculating the percentage of missing data for each item. Missing data of about 1–2% are generally acceptable [13].

#### Floor and ceiling

Floor and ceiling effects were analysed by calculating the proportion of participants scoring at the lowest and highest possible levels. A floor or ceiling effect was considered if at least 50% of the respondents scored at the minimum or maximum level [22].

#### Convergent validity

The convergent validity between the ARNC, QLQ-C30 and DT was evaluated by testing the association between selected items and scales. A total of 20 items in the DT and 24 items and six scales in the QLQ-C30 were used for comparison of 19 items in the ARNC. Five of the ARNC items, *Memory/Focus*, *Physical activity*, *Personal finances*, *Balance* and *Addiction*, have no equivalent in the DT. Two ARNC items, *Balance* and *Addiction*, have no counterpart in the QLQ-C30. Spearman’s rank correlation test was used to test convergent validity and correlations were interpreted as low (< 0.30), medium (0.30–0.49), or strong (≥ 0.50) [23].

#### Known-groups validity

Known-groups analysis was performed to test the sensitivity of the ARNC to capture expected differences between subgroups based on gender, age and education [13]. The assumption was, based on earlier studies [24, 25] that men report better health than women, and less rehabilitation needs b) that physical health gradually deteriorate with age and have more rehabilitation needs d) that those with a low level of education, report poorer health and have more rehabilitation needs.

#### Factor analysis

Exploratory factor analysis (EFA) was conducted to assess the underlying factor structure of the ARNC items. It was hypothesized that two factors, representing physical and mental health symptoms, would be extracted, but that several items would not load on any specific factor. The Kaiser-Meyer-Olkin (KMO) measure of sampling adequacy and Bartlett’s test of sphericity were used to determine whether the data were suitable for factor analysis. The EFA was conducted using principal axis factoring and Promax rotation method. Kaiser criterion (eigenvalue > 1) and interpretability were considered to determine the number of factors to be retained. Items with a minimum loading of 0.40 were considered to contribute to a given factor.

Confirmatory factor analysis (CFA) using maximum likelihood estimation was performed to test the goodness of fit of the factor model. The Satorra–Bentler scaled chi-square (S-Bχ^2^) was applied [26]. The fit of the factor model was evaluated using the comparative fit index (CFI), the Tucker–Lewis index (TLI), the standardized root mean square residual (SRMR) and the root mean square error of approximation (RMSEA). Values ≥ 0.95 for the CFI and TLI, ≤ 0.08 for the SRMR, and ≤ 0.06 for the RMSEA were considered to constitute adequate goodness of fit [27].

## Results

**Fig. 1:**
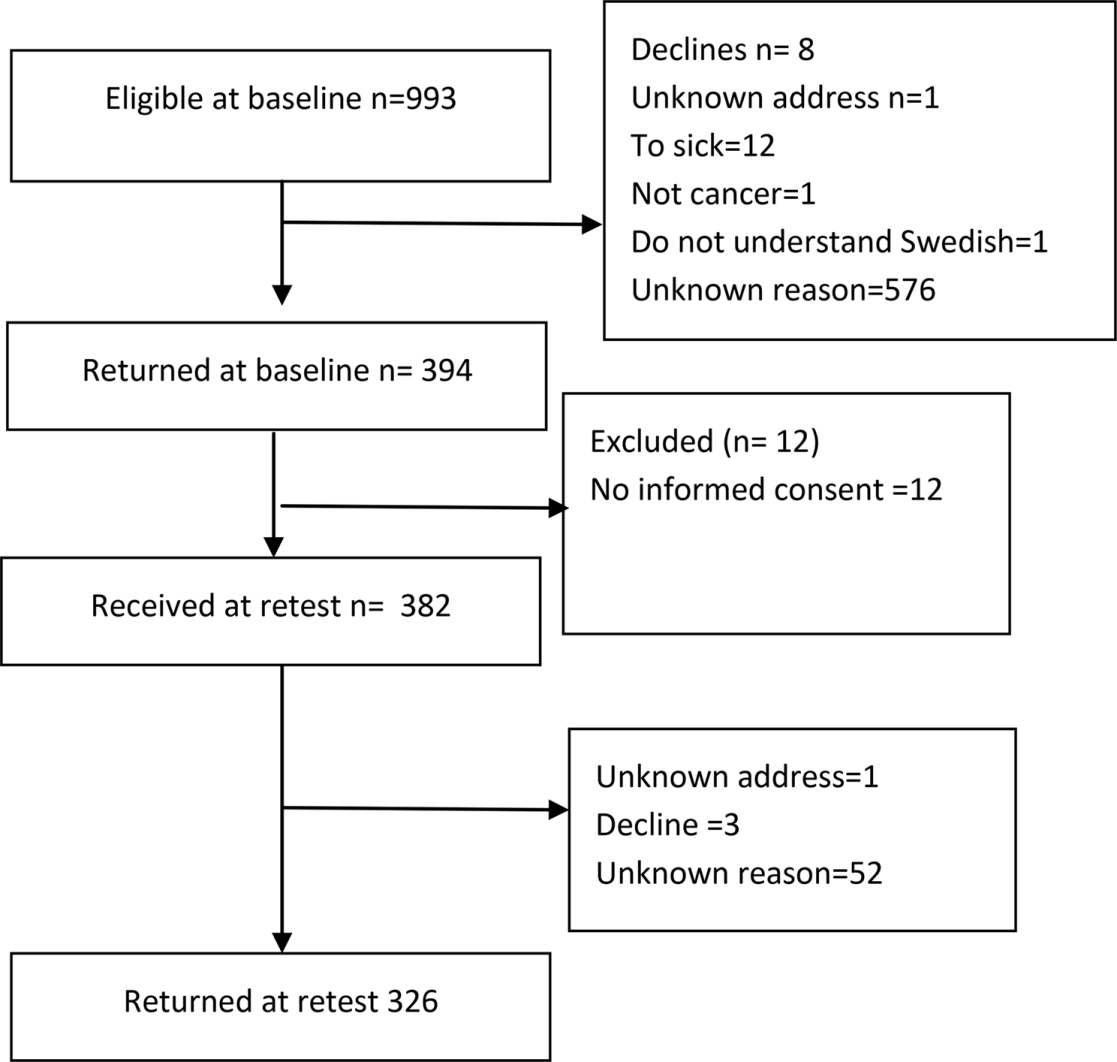
Flow diagram of survey participation

A total of 382 (38%) persons answered the baseline questionnaires and 326 of these (85.3%) completed the retest (Fig. 1). Women constituted 45.1% of the sample (Table 1). The mean (SD) age was 70.4 (10.9) years (range 2297 years). Almost half of the patients (45.5%) were between 70 and 79 years old and 75% were on retirement pension. The large majority were married or cohabiting (69.6%). One-third (36.6%) had a university education, 25% had a high school education, and 30% a mandatory education.

Breast and prostate cancer were the largest diagnostic groups, together making up for 46.8%, followed by lymphatic and blood malignancies, together accounting for 10.2%. All other diagnostic groups made up < 10% of the total sample (Table 1).

### Completeness of data

The percentage of missing data for ARNC items ranged between 1.8% and 8.4% (mean 2.5%). The item about sexuality had the highest percentage of missing data; with this item removed, the mean missing data were 2.1%. The percentage of missing data for items in the DT ranged from 2.1 to 5.2% (mean 3.4%), while in the QLQ-C30, missing data for single items ranged from 1.1 to 2.4% (mean 1.5%).

### Floor and ceiling effects

The proportion of participants selecting the lowest response option (“no problem”) on the ARNC was lowest for fatigue (33.8%) and highest for personal finances (83.0%) (Table 2). A floor effect (≥ 50%) was observed for 17 of the 21 items. The proportion of respondents who chose the lowest response option (“no problem”) on the DT varied between 55.2% for fatigue and 92.7% for the item *God-related existential thoughts*. A floor effect (≥ 50%) was observed for all of the 20 DT items that corresponded to ARNC items. At an item level, the proportion of participants who chose the lowest response level on the QLQ-C30 varied from 30.1% (*Were you tired?*) to 87.7% (*Physical condition/treatment caused financial difficulties*). A floor effect was seen for 16 of the 24 items that corresponded to the ARNC items. The mean proportion answering “no problem” was 81% for DT, 64.3% for ARNC, and 56.7% for QLQ-C30 items. No ceiling effects were seen in any of the instruments.

**Table 2:** Proportion of patients reporting no problems on corresponding items on the ARNC, DT, and QLQ-C30 (*n* = 382)

ARNC		DT		QLQ-C30	
Items 4-point response scale	No problem, %	Items/Problem List Yes-no response scale	No problem, %	Items 4-point response scale	No problem, %
Fatigue	33.8	Fatigue	55.2	10. Need rest	34.3
				12. Felt weak	45.3
				18. Tired	30.1
Sleep	49.5	Sleep	65.2	11. Trouble sleeping	50.8
Pain	55.8	Pain	72.3	9. Had pain	48.7
				19. Pain interfere with daily activities	62.8
Breathing	74.6	Breathing	82.7	8. Short of breath	44.2
Memory/focus	56.3	-	-	20. Difficulty concentrating on things	77.5
				25. Difficulty remembering things	50.3
Mood/depression	59.2	Depression	81.2	24. Feel depressed	56.0
Worry/anxiety	57.3	Worry	80.9	21. Feel tense	66.8
				22. Worry	53.7
Food/drink	84.3	Eating	89.5	13. Lacked appetite	79.6
Nausea	83.2	Nausea	88.7	14. Felt nauseated	82.5
Stool	64.9	Constipation	84.6	16. Been constipated	76.7
		Diarrhea	82.5	17. Had diarrhea	73.3
Physical activity	42.1	-	-	1. Trouble doing strenuous activities	45.6
				2. Trouble talking a long walk	44.5
				3. Trouble taking a short walk outside	77.5
				4. Need to stay in bed or chair during the day	46.3
Family/friends	78.0	*Family problems*:			
		Relationship with the children	90.8	26. Physical condition/treatment interfered with family activity	63.1
		Relationship with the partner	88.5	27. Physical condition/treatment interfered with social activity	58.4
Personal finances	83.0	-	-	28. Physical condition/treatment caused financial difficulties	87.7
Work/voluntary work	71.7	Work/studies	89.8	6. Limited in doing either work or daily activities	61.5
Tingling in hands/feet	62.3	Tingling in hands and feet	69.9		
Urine	68.5	Changes in urination	75.1		
Balance	56.0	-	-		
Appearance	73.0	Appearance	84.6		
Sexuality	46.1	Sexual problems	70.7		
Existential thoughts	63.1	*Existential thoughts*:			
		Lost faith	89.3		
		God related	92.7		
		Life has lost meaning or purpose	86.4		
Addiction	87.4	-	-		

*ARNC* Assessment of Rehabilitation Needs Checklist, *DT* Distress Thermometer, *QLQ-C30* Quality of Life Questionnaire - Core 30 items

### Convergent validity

A strong correlation between the ARNC and DT was observed for two items, a medium correlation for 13 items, and a low correlation for one item (*God-related existential thoughts* in the DR, and *Existential thoughts* in the ARNC) (Table 3). A strong correlation between ARNC and QLQ-C30 scores was found for all functional scales and for 17 of the 24 items. A medium correlation was found for seven items.

**Table 3:** Correlations between corresponding items and scales for ANC, DT and QLQ-30 (*n* = 382)

ARNC	DT	ARNC vs. DT	EORTC -QLQ30	ARNC vs. EORTC
Fatigue	Fatigue	0.60	10. Did you need rest	*0.63*
			12. Have you felt weak	*0.54*
			18. Were you tired?	*0.73*
Sleep	Sleep	0.65	11. Do you have trouble sleeping?	*0.75*
Pain	Pain	0.58	9. Have you had pain?	*0.74*
			19. Did pain interfere with you daily activities?	*0.61*
Breathing	Breathing	0.63	8. Were you short of breath?	*0.57*
Memory/focus			Cognitive function scale	0.70
	-	-	20. Have you had difficulty concentrating on things?	0.46
			25. Have you had difficulty remembering things?	*0.69*
Mood/depression	Depression	0.58	Emotional function scale	0.75
			24. Did you feel depressed?	*0.75*
Worry/anxiety	Worry	0.59	Emotional function scale	0.74
			21. Did you feel tense?	*0.58*
			22. Did you worry?	*0.76*
Food/drink	Eating	0.50	13. Have you lacked appetite	*0.55*
Nausea	Nausea	0.62	14. Have you felt nauseated?	*0.73*
Stool	Constipation	0.47	16. Have you been constipated?	*0.56*
	Diarrhea	0.42	17. Have you had diarrhea	0.40
Physical activity			Physical function scale	0.69
			Role Function scale	0.61
			1. Do you have trouble doing strenuous activities?	*0.55*
			2. Do you have any trouble talking a long walk	*0.66*
			3. Do you have any trouble taking a short walk outside?	0.49
			4. Do you need to stay in bed or chair during the day	0.48
Family/friends	*Family problems*: Relationship with the children	0.33	26. Have your physical condition or medical treatment interfered with family activity?	0.39
	Relationship with the partner	0.44	27. Har Ditt fysiska tillstånd eller den medicinska behandlingen stört Dina sociala aktiviteter?	0.38
Personal finances			28. Have your physical condition or medical treatment caused financial difficulties?	*0.60*
Work/voluntary work	Work/studies	0.36	6. Were you limited in doing either work or daily activities?	0.45
Tingling in hands/feet	Tingling in hands and feet	0.77	-	
Urine	Changes in urination	0.66	-	
Balance	-	-	-	
Appearance	Appearance	0.61	-	
Sexuality	Sexual problems	0.70	-	
Existential thoughts	*Existential thoughts*: Lost faith	0.31	Emotional function scale	0.57
	God related	0.22		
	Life has lost meaning or purpose	0.39		
Addiction	-	-	-	-

*Spearman correlations *p* < 0.001

*ARNC* Assessment of Rehabilitation Needs Checklist, *DT* Distress Thermometer, *QLQ-C30* Quality of Life Questionnaire - Core 30 items

### Factor analysis

Analysis of the 21 ARNC items showed that the KMO test was 0.87 and Bartlett’s test of sphericity *p* < 0.001, indicating that the data were suitable for factor analysis. The EFA showed a two-factor model that explained 85% of the common variance (Supplementary Table A). The first factor contained eight items with loadings of ≥ 0.40 (*Balance*, *Physical activity*, *Fatigue*, *Pain*, *Tingling in hands/feet*, *Stools*, *Breathing*, and *Memory/focus*) and was interpreted to constitute a physical health symptom factor. The second factor included five items (*Mood/depression*, *Worry/anxiety*, *Existential thoughts*, *Family/friends*, and *Appearance*) and was considered a mental health symptom factor. A total of eight items did not load on either of the two factors (*Sleep*, *Food/drink*, *Nausea*, *Work/voluntary work*, *Sexuality*, *Urine*, *Addiction*, and *Personal finances*).

Confirmatory factor analysis of the 13-item two-factor model showed a RMSEA value of 0.04, CFI and TLI values of 0.97 and 0.96, respectively, and a SRMR value of 0.05, indicating satisfactory model fit (Supplementary Table B).

### Test–retest reliability

As previously mentioned, the retest was answered by 326 (85.3%) persons. The test–retest analysis showed that the ICC was ≥ 0.80 for twelve of the ARNC items, and between 0.70 and 0.79 for eight items, while one item (*Addiction*) was below 0.70 (Supplementary Table C).

### Reported cancer-related problems

For the total sample, problems with the highest rates were reported for *Sexuality*, *Fatigue*, *Physical activity*, *Sleep*, and *Pain* (Table 4).

**Table 4:** ARNC item mean (0-100 scale) and standard deviation (SD) by gender

ARNC items	Total (*n* = 377)	Men (*n* = 209)	Women (*n* = 172)	Men vs. women	Men vs. women
	Mean (SD)	Mean (SD)	Mean (SD)	*p*-value*	Effect size**
Fatigue	30.1 (27.5)	25.3 (25.6)	35.9 (28.7)	< 0.001	0.39
Sleep	23.4 (27.2)	19.3 (24.9)	28.2 (29.1)	0.003	0.33
Pain	20.3 (27.1)	16.2 (25.2)	25.1 (28.4)	< 0.001	0.33
Breathing	10.1 (20.1)	8.9 (19.0)	11.6 (21.2)	0.201	0.13
Memory/focus	17.3 (22.3)	14.7 (21.7)	20.5 (22.7)	0.006	0.26
Mood/depression	17.6 (24.5)	14.6 (23.4)	21.2 (25.3)	0.004	0.27
Worry/anxiety	17.9 (24.3)	14.2 (22.7)	22.3 (25.5)	0.000	0.34
Food/drink	5.8 (15.4)	4.4 (14.7)	7.4 (16.1)	0.011	0.19
Nausea	5.8 (15.0)	3.8 (10.6)	8.2 (18.7)	0.017	0.29
Stool	14.5 (23.2)	14.3 (22.2)	14.7 (24.4)	0.785	0.02
Physical activity	26.5 (27.6)	23.6 (26.4)	30.0 (28.7)	0.025	0.23
Family/friends	8.2 (18.2)	8.5 (19.2)	7.7 (17.1)	0.789	0.04
Personal finances	6.8 (17.8)	5.8 (16.7)	8.1 (19.1)	0.166	0.13
Work/voluntary work	12.5 (23.5)	10.6 (22.2)	14.9 (24.9)	0.064	0.18
Tingling in hands/feet	18.1 (28.1)	17.1 (26.4)	19.3 (30.1)	0.750	0.08
Urine	13.1 (22.4)	18.0 (24.4)	7.2 (18.3)	< 0.001	0.50
Balance	18.0 (24.2)	17.5 (23.7)	18.5 (24.9)	0.788	0.04
Appearance	11.3 (21.5)	7.3 (18.5)	16.1 (23.8)	< 0.001	0.41
Sexuality	30.5 (36.5)	39.1 (38.7)	19.6 (30.3)	< 0.001	0.56
Existential thoughts	15.6 (24.1)	12.3 (21.8)	19.5 (26.0)	0.002	0.30
Addiction	5.0 (16.5)	3.9 (15.8)	6.2 (17.4)	0.096	0.14

*ARNC* Assessment of Rehabilitation Needs Checklist, *SD* Standard deviation

*Mann-Whitney U-test

**Effect size criteria: <0.20 = trivial, 0.20–0.49 = small, 0.50–0.79 = medium, and ≥ 0.80 = large

#### Gender

There was a significant difference between men and women for 13 of the 21 ARNC items (Table 4). Women reported more problems than men on eleven items (*Fatigue*, *Sleep*, *Pain*, *Memory/focus*, *Mood/depression*, *Worry/anxiety*, *Food/drink*, *Nausea*,* Physical activity*, *Appearance*, and *Existential thoughts*). Men reported more problems regarding *Sexuality* and *Urine.*

#### Age groups

Compared with the oldest group (70 + years), participants in the 22–69 age group reported significantly more problems regarding W*orry/anxiety*, *Personal finances*, *Work/voluntary work*, *Appearance*, and *Existential thoughts* (Table 5). Participants aged ≥ 70 years reported significantly more problems related to *Stools*, *Urine*, and *Balance* than did those aged 22–69 years.

**Table 5:** ARNC item mean (0-100 scale) and standard deviation (SD) by age and education

ACRN items	Total (*n* = 377)	Age 29–69 year	Age year 70+	Younger Vs Older	Younger Vs Older	Manda tory	Higher education	Mandatory Vs Higher ed	Mandatory Vs Higher ed
	Mean (SD)	Mean (SD)	Mean (SD)	*p*-value*	Effect size**	Mean (SD)	Mean (SD)	*p*-value*	Effect size**
Fatigue	30.1 (27.5)	30.2 (28.3)	30.1 (27.1)	0.988	0.00	30.4 (27.8)	30.1 (27.5)	0.975	0.01
Sleep	23.4 (27.2)	25.4 (28.3)	22.1 (26.5)	0.285	0.12	22.9 (25.3)	23.5 (28.1)	0.891	0.02
Pain	20.3 (27.1)	19.0 (26.4)	21.1 (27.5)	0.490	0.08	23.2 (28.6)	18.9 (26.2)	0.167	0.16
Breathing	10.1 (20.1)	10.1 (19.1)	10.1 (20.7)	0.798	0.00	14.2 (24.5)	8.3 (17.6)	0.021	0.28
Memory/focus	17.3 (22.3)	17.5 (22.8)	17.2 (22.1)	0.975	0.03	18.0 (21.0)	17.0 (22.9)	0.410	0.04
Mood/depression	17.6 (24.5)	18.6 (25.5)	17.0 (23.9)	0.651	0.06	15.2 (20.9)	18.7 (25.8)	0.420	0.15
Worry/anxiety	17.9 (24.3)	22.0 (25.9)	15.5 (23.1)	0.010	0.26	16.5 (22.0)	18.6 (25.3)	0.678	0.09
Food/drink	5.8 (15.4)	7.2 (16.0)	4.9 (15.0)	0.054	0.15	5.1 (15.6)	6.1 (15.3)	0.365	0.06
Nausea	5.8 (15.0)	7.0 (17.7)	5.1 (13.1)	0.520	0.12	4.8 (11.7)	6.2 (16.2)	0.688	0.15
Stool	14.5 (23.2)	10.4 (21.2)	16.9 (23.9)	0.002	0.29	16.1 (22.0)	13.9 (23.7)	0.126	0.10
Physical activity	26.5 (27.6)	23.5 (26.8)	28.2 (28.1)	0.099	0.17	25.9 (27.5)	26.4 (27.5)	0.828	0.02
Family/friends	8.2 (18.2)	10.6 (20.5)	6.7 (16.6)	0.035	0.21	6.8 (16.3)	8.8 (19.0)	0.341	0.11
Personal finances	6.8 (17.8)	12.4 (23.2)	3.6 (12.8)	< 0.001	0.47	6.9 (20.7)	6.9 (16.6)	0.378	0.00
Work/voluntary work	12.5 (23.5)	19.0 (28.2)	8.8 (19.4)	< 0.001	0.42	11.7 (22.8)	12.7 (23.7)	0.726	0.03
Tingling in hands/feet	18.1 (28.1)	18.4 (27.8)	17.9 (28.4)	0.794	0.02	20.0 (29.0)	17.1 (27.7)	0.298	0.10
Urine	13.1 (22.4)	8.4 (18.0)	15.8 (24.3)	0.001	0.35	16.5 (23.7)	11.7 (21.8)	0.019	0.21
Balance	18.0 (24.2)	12.5 (21.0)	21.2 (25.5)	0.003	0.37	21.4 (24.0)	16.1 (23.7)	0.015	0.22
Appearance	11.3 (21.5)	16.4 (24.2)	8.3 (19.2)	0.001	0.37	6.8 (17.4)	13.3 (22.9)	0.005	0.32
Sexuality	30.5 (36.5)	28.9 (34.7)	31.5 (37.7)	0.730	0.07	33.3 (36.8)	29.1 (36.3)	0.226	0.12
Existential thoughts	15.6 (24.1)	19.7 (26.3)	13.2 (22.4)	0.010	0.27	11.1 (19.8)	17.6 (25.5)	0.022	0.28
Addiction	5.0 (16.5)	3.6 (13.2)	5.7 (18.2)	0.376	0.132	4.8 (14.9)	5.0 (17.3)	0.682	0.01

*ARNC* Assessment of Rehabilitation Needs Checklist

*Mann-Whitney U-test

**Effect size criteria: <0.20 = trivial, 0.20–0.49 = small, 0.50–0.79 = medium, and ≥ 0.80 = large. Cohen’s d

#### Educational level

Those with mandatory education reported significantly larger problems with *Breathing*, *Urine* and *Balance* compared with those with higher education, while those with higher education reported significantly larger problems with *Appearance* and *Existential thoughts* (Table 5).

## Discussion

In this study, we evaluated the psychometric properties of the ARNC, an instrument developed to screen cancer survivors for health problems that may require rehabilitation interventions. The ARNC demonstrated acceptable reliability and validity.

Estimates of test–retest reliability were adequate for all ARNC items except for *Addiction.* The low test–retest value for *Addiction* is an indication that the item is too vague and in need of an explanation, mainly regarding overuse of drugs, alcohol or tobacco. Another option is to remove the item from the ARNC as a result of the validation process.

As expected, substantial floor effects at the item level were observed for all three instruments; however, the proportion reporting no problems varied between the instruments. The agreement between the ARNC and the QLQ-C30 was generally satisfactory, while the floor effects for the DT were considerably larger. This is probably an effect of the dichotomized yes/no response options used in the DT, while the ARNC and QLQ-C30 use 4-point response options. These findings indicate that the DT has a weaker ability to detect health problems compared with the ARNC and QLQ-C30. The DT also has limited ability to detect progression over time since there are no scale steps between yes and no. The DT’s ability to monitor changes over time in global distress (0–10 response scale) was good, according to a previous validation of the Swedish DT, but corresponding changes for the 35-item “problem list” were not reported [18].

The completeness of data was satisfactory for the ARNC, indicating that the questionnaire was well accepted by the respondents. The item asking about problems with sexuality showed most missing data (32%). A low response rate for items concerning sexuality has been observed in many previous studies [25, 28, 29]. Of all three instruments the DT had the highest proportion of missing data, ranging from 8 to 20%. The reason may be that respondents only answer items that they have problems with and leave other items unanswered, as suggested in a study where the Swedish version of the DT “problem list” was validated [19].

The correlations between ARNC items and items and scales in the well-established QLQ-C30 and DT were medium to strong, indicating acceptable convergent validity. Only the ARNC item *Existential thoughts* correlated below 0.30 to the DT item *God-related thoughts*.

Known-groups analysis indicated that the ARNC items are sensitive to detect relevant differences between subgroups based on gender, age and education. Men reported significantly lower levels of problems than women on eleven out of 21 items, which is similar to other studies of HRQoL in cancer populations [15, 25, 30].

In this study, physical symptoms were a greater problem among those with only mandatory education, while participants with a higher education reported more distress regarding the psychological problems *Appearance* and *Existential thoughts*. The result is similar to a Danish study that reported higher risk of impaired functioning and severe symptoms in persons with lower education compared with those with higher education. The differences could persist as long as 12 years after diagnosis [31]. Higher education was an independent predictor of better physical QoL in a recent international survey by Maxwell et al. [32].

The participants aged 70 years and older reported more problems with *Stools*, *Urine*, and *Balance* compared with younger participants, which is expected as these problems increase with older age. Constipation is common among the elderly, with a prevalence of about 20% [33]. Younger patients aged 22–69 years reported more problems with *Personal finances* compared with older patients. This difference is expected as sickness benefits are lower than the usual salary. Retired persons in Sweden can depend on their pension. Most of the treatment costs for cancer patients are covered by the social security system, which may explain the low proportion of respondents reporting problems with personal finances. This result may differ from studies in countries with different social security systems, where financial toxicity is reported as a common problem [34].

Factor analysis of the 21 ARNC items confirmed the hypothesized two-factor structure with a physical and a mental health symptom factor. The two factors comprised eight and five items, respectively, while eight items did not load on either factor. These factors may be used as multi-item scales if more comprehensive measures of patients’ health are required, especially for measuring change over time. The multi-item scales may also be used for research purposes.

The highest proportion of reported problems in our study was observed for *Sexuality*, *Fatigue*, *Physical activity*, *Sleep*, and *Pain.* This is similar to a Swedish study from 2013 [18] where *Fatigue*, *Sleep*, *Pain*, and *Sexual problems* were among the most frequently reported problems. Symptom clusters similar to the most common problems in our study have also been found elsewhere in previous studies. These clusters have consisted of various combinations of *Fatigue*, *Depression*, *Anxiety*, *Pain*, *Sleep disturbance*, and *Cognitive dysfunction* [35]. Fatigue is the most frequently reported symptom among cancer survivors [35–37]. In this study, a total of 66.2% (ARNC), 65.7% (QLQ-C30) and 44.8% (DT) reported problems with fatigue. The low percentage for the DT can be explained by the instrument’s dichotomous response option and a higher threshold for reporting “yes”, which produces large floor effects.

*Mood/Depression* and *Worry/Anxiety* were reported as “no problem” by > 80% of the respondents in our study, but seem more common in other studies [35, 38, 39]. This difference could be explained by differences in the samples, as most participants in our study had started or even finished their treatment, while the sample in Thalen-Lindstrom’s study [10] were recruited at the first visit to the oncologist. It is possible that as cancer treatments have become more effective, with high survival rates, symptoms of anxiety and depression are less prominent.

A strength of this study is that a wide spectrum of cancer diagnoses was included and that the participants were recruited from six regions in Mid-Sweden, and included both participants who were being treated in a small hospital and participants treated in large hospitals.

One weakness of the study is the low response rate (38%). The proportion of participants in Swedish population surveys has declined in recent decades [24, 40] and a low response rate has been seen also in studies of cancer populations [15, 41]. An other limitation is that the population is skewed towards older ages although that might not influence the psychometric evaluation but might have an impact on norm values. Most cancer diagnosis is received after the age of 70 years. An alternative study design would have been to consecutively include participants at cancer clinics instead of recruiting them through a register. This would have provided the opportunity to include patients at an earlier stage of the disease, compared with the present study population who responded 6–12 months after being diagnosed. However, the interest of Swedish cancer patients in participating in cancer rehabilitation activities tends to be higher after 12 months than in the earlier stages of the disease [15]. Despite the low response rate the aim of the study – to test the validity of the ARNC by comparing it with the DT and QLQ-C30 – was still accomplished as the large sample reported a wide range of responses.

## Conclusions

Our findings suggest that the ARNC is an acceptable and reliable screening instrument for detecting symptoms and signs indicating a possible need for rehabilitation. ARNC is a single-item questionnaires, that is considered easy to implement in clinical care [42]. The ARNC should be used as a first step accompanied by a clinical follow-up consultation focusing on the patient’s health problems and appropriate rehabilitation efforts.

Our findings further indicate that the accuracy of the ARNC is reasonable as a first-stage screening tool to detect symptoms and signs among cancer patients. The ARNC can help contact nurses and other health care personnel to systematically assess the need for rehabilitation and guide the patients to self-care or advanced cancer rehabilitation.

The medium to strong correlations between ARNC items and QLQ-C30 items and scales suggest that the ARNC could be an alternative also for research purposes when a easier and less comprehensive instrument is needed. The simple design could be an advantage as it lowers the burden on cancer patients.

The ARNC was primarily developed for use in people diagnosed with cancer, but the instrument’s wording does not specifically include the word “cancer”. The ARNC could therefore be considered for use in other populations with health problems, after a proper validation.

## Electronic supplementary material

Below is the link to the electronic supplementary material.

Supplementary Material 1

Supplementary Material 2

Supplementary Material 3

Supplementary Material 4

## Data Availability

The data that support the findings of this study are available from the University Health Care Research Centre, Faculty of Medicine and Health, Örebro University, Örebro, Sweden, but restrictions apply to the availability of data which were used under license for the current study, and therefore are not publicly available.

## Abbreviations

ARNC: Assessment of Rehabilitation Needs Checklist
CFA: Confirmatory factor analysis
CFI: Comparative fit index
DT: Distress Thermometer
EFA: Exploratory factor analysis
EORTC: European Organization for Research and Treatment of Cancer
ES: effect size
HRQoL: Health-related quality of life
ICC: Intraclass correlation coefficient
KMO: Kaiser-Meyer-Olkin (test)
QLQ-C30: European Organization for Research and Treatment of Cancer Quality of Life Questionnaire
QoL: Quality of life
RMSEA: Root mean square error of approximation
S-Bχ^2^: Satorra–Bentler scaled chi-square
SD: Standard deviation
SRMR: Standardized root mean square residual
TLI: Tucker–Lewis index
