# Supplementary material for: Validation of the Assessment of Rehabilitation Needs Checklist in a Swedish cancer population
Source: J Patient Rep Outcomes. 2024 Dec 5;8:142. doi: 10.1186/s41687-024-00818-5 (PMC11621288; doi:10.1186/s41687-024-00818-5)
Supplement: Supplementary file 2 — Supplementary Material 2 [file 41687_2024_818_MOESM2_ESM.docx]

Supplementary table X. Exploratory factor analysis of the 21-item ARNC (n=382).

|  | **Factor loadings**^a^ | |
| --- | --- | --- |
| **ARNC items** | **Factor 1**  **Physical** | **Factor 2**  **Mental** |
| Eigenvalue^b^ | 5.89 | 1.13 |
| Common variance explained^c^ | 71% | 14% |
| *Physical symptoms*^d^ |  |  |
| Balance | **0.78** | -0.23 |
| Physical activity | **0.74** |  |
| Fatigue | **0.65** |  |
| Pain | **0.56** |  |
| Tingling in hands/feet0. | **0.50** |  |
| Stool | **0.51** |  |
| Breathing | **0.43** |  |
| Memory/focus | **0.41** | 0.24 |
| *Mental symptoms*^d^ |  |  |
| Mood/depression |  | **0.90** |
| Worry/anxiety |  | **0.89** |
| Existential thoughts |  | **0.78** |
| Family/friends |  | **0.52** |
| Appearance |  | **0.62** |
| *Individual symptoms*^e^ |  |  |
| Sleep | 0.36 | 0.28 |
| Food/drink | 0.23 | 0.21 |
| Nausea | 0.23 |  |
| Work/voluntary work | 0.29 | 0.28 |
| Sexuality | 0.31 |  |
| Urine | 0.34 |  |
| Addiction | 0.24 |  |
| Personal finances |  | 0.36 |

^a^Pattern matrix; standardized regression coefficients; factor loadings ≥ 0.40

are in bold; loadings < 0.20 are not shown.

^b^Factors with an eigenvalue > 1 were extracted (Kaiser's criterion).

^c^Total common variance explained by the two factors: 50% = acceptable; ≥70% = good.

^d^Items that load (≥ 0.40) on the physical or mental factor.

^e^Items that do not load (< 0.40) on the physical or mental factor.
