# Supplementary material for: Validation of the Assessment of Rehabilitation Needs Checklist in a Swedish cancer population
Source: J Patient Rep Outcomes. 2024 Dec 5;8:142. doi: 10.1186/s41687-024-00818-5 (PMC11621288; doi:10.1186/s41687-024-00818-5)
Supplement: Supplementary file 3 — Supplementary Material 3 [file 41687_2024_818_MOESM3_ESM.docx]

Supplementary table B. Confirmatory factor

analysis of the ARNC 13-item two-factor model.

| *Measures of model fit* | |
| --- | --- |
| df | 64 |
| Normal theory x^2^ | 138.53 |
| S-Bx^2^ | 93.61 |
| S-Bx^2^/df | 1.46 |
| RMSEA | .037 |
| CFI | .971 |
| TLI | .964 |
| SRMR | .052 |

df = degrees of freedom

x^2^ = chi-square

S-Bx^2^ = Satorra–Bentler scaled chi-square

RMSEA = root mean square error of approximation

CFI = comparative fit index

TLI = Tucker–Lewis index/non-normed fit index

SRMR = standardized root mean square residual
