# Supplementary material for: Validation of the Assessment of Rehabilitation Needs Checklist in a Swedish cancer population
Source: J Patient Rep Outcomes. 2024 Dec 5;8:142. doi: 10.1186/s41687-024-00818-5 (PMC11621288; doi:10.1186/s41687-024-00818-5)
Supplement: Supplementary file 4 — Supplementary Material 4 [file 41687_2024_818_MOESM4_ESM.docx]

Table 4. Test-retest reliability. Intraclass correlation coefficients (ICC) for ARNC items.

| **ARNC items** | **ICC (95% CI)** |
| --- | --- |
| Fatigue | 0.82 (0.78 – 0.86) |
| Sleep | 0.81 (0.77 – 0.85) |
| Pain | 0.79 (0.74 – 0.83) |
| Breathing | 0.84 (0.80 – 0.87) |
| Memory/focus | 0.81 (0.76 – 0.85) |
| Mood/depression | 0.87 (0.84 – 0.90) |
| Worry/anxiety | 0.86 (0.83 – 0.89) |
| Food/drink | 0.73 (0.67 – 0.78) |
| Nausea | 0.72 (0.65 – 0.78) |
| Stool | 0.79 (0.73 – 0.83) |
| Physical activity | 0.79 (0.73 – 0.83) |
| Family/friends | 0.73 (0.66 – 0.78) |
| Personal finances | 0.84 (0.79 – 0.87) |
| Work/voluntary work | 0.73 (0.66 – 0.78) |
| Tingling in hands/feet | 0.88 (0.85 – 0.91) |
| Urine | 0.80 (0.76 – 0.84) |
| Balance | 0.84 (0.80 – 0.87) |
| Appearance | 0.79 (0.73 – 0.83) |
| Sexuality | 0.87 (0.84 – 0.90) |
| Existential thoughts | 0.85 (0.82 – 0.88) |
| Addiction | 0.62 (0.52 – 0.69) |

95% CI=confidence interval
